# Emergence of the ability to perceive dynamic events from still pictures in human infants

**DOI:** 10.1038/srep37206

**Published:** 2016-11-17

**Authors:** Nobu Shirai, Tomoko Imura

**Affiliations:** 1 Department of Psychology, Faculty of Humanities, Niigata University, 2-8050 Ikarashi Nishi-Ku Niigata, 950-2181, Japan; 2 Department of Information Systems, Faculty of Information Culture, Niigata University of International and Information Studies, 3-1-1, Mizukino, Nishi-ku, Niigata, 950-2292, Japan

## Abstract

The ability to understand a visual scene depicted in a still image is among the abilities shared by all human beings. The aim of the present study was to examine when human infants acquire the ability to perceive the dynamic events depicted in still images (implied motion perception). To this end, we tested whether 4- and 5-month-old infants shifted their gaze toward the direction cued by a dynamic running action depicted in a still figure of a person. Results indicated that the 5- but not the 4-month-olds showed a significant gaze shift toward the direction implied by the posture of the runner (Experiments 1, 2, and 3b). Moreover, the older infants showed no significant gaze shift toward the direction cued by control stimuli, which depicted a figure in a non-dynamic standing posture (Experiment 1), an inverted running figure (Experiment 2), and some of the body parts of a running figure (Experiment 3a). These results suggest that only the older infants responded in the direction of the implied running action of the still figure; thus, implied motion perception emerges around 5 months of age in human infants.

The fact that even ancient humans had the ability to create concrete drawings (cf.^1^) demonstrates that the ability to express (or perceive) particular visual scenes based on still images is a fundamental aspect of human nature. On the one hand, humans perceive and understand objects or events depicted in still images very easily; on the other hand, however, there are considerable individual differences in drawing visual scenes as still images. These everyday observations imply that the ability to “see” particular objects or events in a static figure seems to be more common than the ability to draw (elaborated) figures among human beings. Indeed, one of the basic contributors to the ability to “see” still images can be observed even in infants who cannot draw. For instance, infants can perceive three-dimensional structures in visual scenes based on particular visual information contained in still images (pictorial depth cues) even in their first 6 months e.g. refs 2 and 3.

Moreover, infants and toddlers seem to understand the meanings of the dynamic events that are depicted in still images. Several studies have shown that children aged as young as 4 years can understand dynamic motor actions when they are expressed in the posture assumed by a cartoon character in a still image^4^,^5^. Additionally, a recent study^6^ demonstrated that even 5–8-month-old infants significantly shifted their gaze toward the direction of a dynamic action depicted in a still image. Such responses in infants and toddlers, to dynamic events depicted in a still image, may be comparable to adults’ perceptions of directional motion based on non-directional static images (implied motion perception)^7^.

One open question about the development of implied motion perception concerns when human beings acquire this ability during ontogeny. The aim of the present study was to explore the developmental origin of implied motion perception in human beings. To this end, we tested 4- and 5-month-old infants. These age periods were chosen based on previous findings on the potential neural bases for implied motion perception and the functional development of such neural bases. The perception of dynamic events based on static figures seems to be related to interactions between the ventral and dorsal visual pathways, which are sensitive to form and motion-related visual information, respectively. For instance, patterns of static forms that imply dynamic events activate motion-sensitive regions, such as the hMT/MST+ in the dorsal pathway^8^,^9^,^10^,^11^,^12^. More directly, it has been shown that motion perception based on non-directional visual stimuli (e.g., dynamic Glass patterns^13^) is correlated with the interactive neural activations between the ventral (V4 and lateral occipital cortex [LOC]) and dorsal (hMT+/V5, V3, V3a, and kinetic occipital [KO] region) streams^14^. Significant behavioral and neural sensitivity to global motion (related to the dorsal pathway) and global form (related to the ventral pathway) patterns develop by 3 and 5 months of age, respectively^15^. This means that functional interactions between the ventral and dorsal pathways appear around 5 months of age. In this context, it is reasonable to infer that implied motion perception emerges by 5 months of age, which links it to the functional development of the interactions between the ventral and dorsal visual pathways. On the other hand, despite these implications, no empirical examinations of whether implied motion perception emerges at around 5 months have been conducted thus far. Thus, the primary aim of the present study was to investigate the developmental onset of implied motion perception among infants aged 4 and 5 months. We found that the fundamental ability to perceive and understand dynamic events from still images develops in human infants at the age of 5 months, concurrent with the maturation of relevant neural regions such as the dorsal and ventral visual pathways.

## General Methods

### Ethics statement

All experiments were approved by the Ethics Committee for Psychological Research at Niigata University and were performed in accordance with the Helsinki Declaration.

### Participants

All infants who participated in this study were recruited from the infant laboratory participant database at the Department of Psychology, Niigata University (Niigata, Japan). Flyers were delivered to the families of the infants via public health service centers in Niigata City, and the infants whose families voluntarily contacted the infant laboratory were registered in the database. Written informed consent was obtained from the parents of the infants before starting the experiments.

### Apparatus

We used PsychoPy2 software^16^ on a laptop computer (MacBook Pro 2300/15 MD103J/A; Apple Inc., Cupertino, CA, USA) to run the experiments. Visual stimuli were presented on a 22-inch CRT display (RDF223H; Mitsubishi, Tokyo, Japan; refresh rate = 60 Hz, resolution = 1024 × 768 pixels, full-color mode) placed in a dark experimental booth. Two loudspeakers placed behind the CRT emitted “beep” sounds to attract infants’ attention to the CRT at the beginning of each experimental trial. Infants sat on their parents’ lap in front of the CRT, and the viewing distance was maintained at approximately 40 cm throughout the experiments. A charge-coupled device (CCD) camera was attached just below the CRT, and video signals from the CCD were sent to a TV monitor placed outside the experimental booth so that an experimenter could observe the infants’ looking behavior on the monitor in real time. The video signals were also sent to a video recorder and recorded as digital video movies.

### Procedures

**Figure 1:**
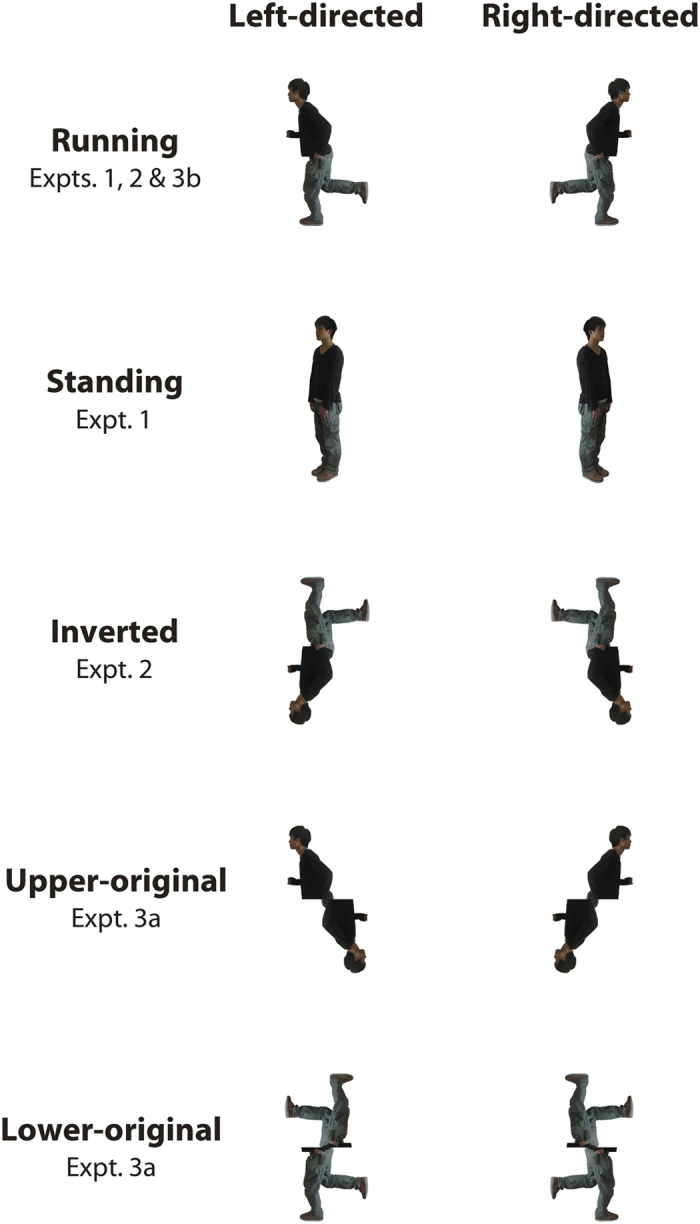
Snapshots of cue figures used in the present study.

The experimental procedure for all experiments in this study was nearly identical to that followed by Shirai and Imura^6^ the exception was our use of a cue figure for a limited duration (600 ms), as the cue disappeared before the presentation of the target discs (see Fig. 1). In preliminary observations, we had found that 4-month-old infants often seemed to experience difficulty shifting their gaze from a cue figure to targets when they were tested with the identical procedure used by Shirai and Imura^6^. It has been established that young infants seem to have difficulty releasing their gaze from an object and shifting it to a competing object (cf. 17). Hence, we limited the presentation time of a cue figure to eliminate the competition between a cue figure and the targets.

**Figure 2:**
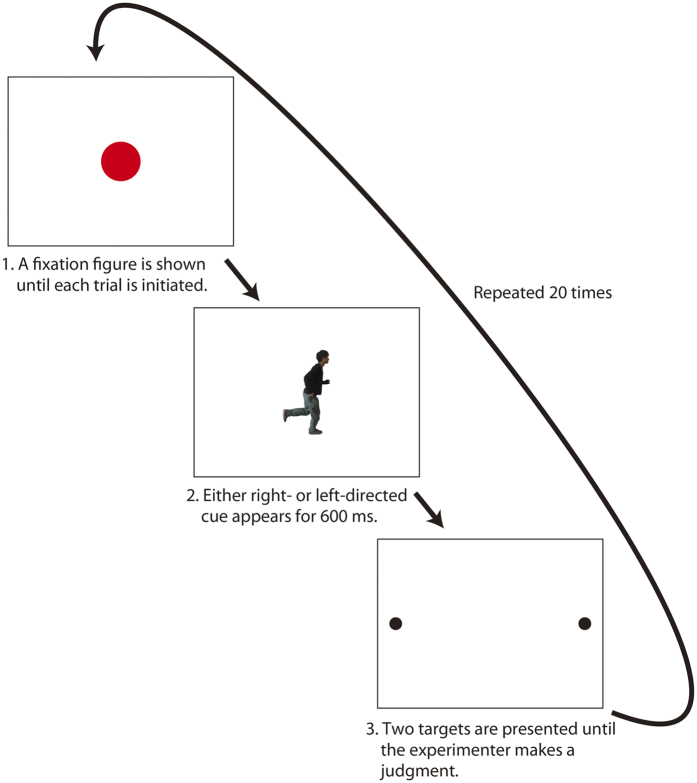
A schematic illustration of the experimental procedure.

Each trial started with the presentation of a colorful cartoon figure accompanied by beep sounds. The figure appeared at the center of a white presentation field (width = 57.3 deg., height = 42.9 deg) until the infant looked at the cartoon. An experimenter then presented a cue figure at the center of the presentation field (see Fig. 2 and the Stimuli section of each experiment for details) for 600 ms; this was immediately followed by two targets (black discs, diameter = 2.5 deg each) that appeared on the presentation field side by side. The distance between the center of each target and the center of the presentation field was 18.5 deg. After the target presentation, the experimenter judged, in real time, the direction of the infant’s first gaze (left or right) by pressing one of two corresponding keys on a personal computer (the forced-choice preferential looking technique^18^). The experimenter was naïve to the experimental trials in each experimental session. When the direction of the infant’s gaze was consistent with the direction of the cue figure, the trial was categorized as a consistent trial. Each experimental condition was composed of 20 experimental trials (two cue figure directions, each repeated 10 times). The presentation order of experimental trials was randomized for each infant. Each infant was tested under two experimental conditions in Experiments 1, 2, and 3a. A rest period (typically >15 min) was provided between sessions to allow parents to feed, change diapers, and play with the infants so they were relaxed and refreshed for the next session.

A preference score for cued direction, i.e., the ratio of consistent trials to total trials under each experimental condition, was calculated for each infant. When the direction of a cue figure affected the infant’s gaze behavior, the mean preference score across infants would be greater than chance (0.5). Inter-observer agreement regarding judgments of infant looking behaviors was tested for 10 randomly chosen experimental sessions (i.e., a total 200 trials) in each experiment. A coder who was naïve to the experimental conditions and purpose of the present study judged infants’ looking behaviors on offline videos. The experimenter and the naïve coder agreed regarding about 98.0% of the trials in Experiment 1, 95.5% of the trials in Experiment 2, 98.5% of the trials in Experiment 3a, and 94.5% of the trials in Experiment 3b.

We should note that there were several differences between the experimental procedures used in the current experiments and in previous studies examining infants’ gaze shift to particular visual stimuli. In previous studies, modified versions of Posner’s cuing paradigm^19^ were typically used to examine gaze shift and the abilities relevant to visual attention in infancy (e.g. refs 20, 21, 22). The differences in gaze latency toward a single target under valid/invalid cued conditions were used as a main dependent variable. On the other hand, in the current study (and our previous study^6^), two targets appeared simultaneously under both the cued and uncued conditions, and the dependent variable was the rate of the congruent first gaze to the cued direction. This somewhat “extraordinary” procedure was inspired by several previous studies^23^,^24^ investigating infants’ gaze behaviors in response to a model’s action displayed as a point-light figure (biological motion figure). In these previous studies, two identical visual targets were placed at both the right and the left sides of a point-light actor, and the rate of the first gaze toward the target corresponding to the direction of the actor’s action was used as the dependent variable. The procedures of the previous studies seemed to work very well, and the aim of the current study, which examined infants’ gaze patterns in response to an actor’s actions, was similar to those of previous studies. Hence, we decided to use an experimental procedure similar to that used by the previous studies.

Additionally, we did not treat the latency of the first gaze as a dependent variable because it was reasonable to assume that the saccadic latencies of the younger and the older infants in our study would significantly differ. Indeed, saccadic latency generally decreases with age in early infancy^25^ and later developmental stages^26^. Such a developmental transition in saccadic latency might obscure differences between the young and older infants in the latency of the first saccade to the cued direction. Even if there were statistically significant differences between younger and older infants in the latency of the first saccade, the differences could be attributable to simpler factors, such as differences in the oculomotor development of the two infant groups, rather than more cognitively-oriented and complex abilities, such as the perception of implied motion. Taking these factors into consideration, we used the rate of the first gaze toward the cued direction (but not the latency) as the dependent variable in the current study.

### Statistical analyses

We performed two different statistical analyses regarding infants’ visual preferences. First, we tested the statistical significance of the infants’ visual preference using a two-tailed t-test and a binomial test against the level of chance (0.5). Preferential looking studies are interested primarily in whether infants show a significant visual preference for one particular visual stimulus over another. It is assumed that, when infants show a significant visual preference for one stimulus, they are sensitive enough to discriminate between visual stimuli by some means. Thus, in the current experimental settings, significantly different preferences (i.e., those less than or greater than 0.5) may reflect significant sensitivity to the cued direction. Second, we performed ANOVAs to examine whether there were any significant differences in visual preferences according to experimental condition and/or age group. As a *t-*test or binomial test against the level of chance would not address the direct difference among preferences under different experimental conditions, we used ANOVA to compare the data between experimental conditions to detect differences in visual preferences by condition and/or age group. Individual data for the current study are available at “ https://nyu.databrary.org/volume/270”.

## Experiment 1

The first aim of Experiment 1 was to replicate the results of Shirai and Imura^6^, who reported that infants aged at least 5 months showed a significant preference for the cued direction when a figure representing a person running was displayed. We used a slightly modified version of their experimental procedure (see General Methods section). A still image, which represents the implied action of a model person, can draw the visual attention of adults to the direction of implied action^26^. Hence, we would expect that the gaze of infants would shift toward the direction of an implied running action if the infants could perceive implied motion from a running figure. The second aim was to test whether 4-month-old infants also showed a visual preference similar to that observed in 5-month-olds.

### Methods

#### Participants

The data were obtained from 20 4-month-old (mean age = 124.2 days, SD = ±9.4 days) and 20 5-month-old (mean age = 155.0 days, SD = ±8.9 days) infants. All infants were healthy, full-term babies and weighed >2,500 g at birth. An additional seven infants also participated in the experiment but were not included in the final sample because they could not complete all experimental trials due to crying (4), fussing (2), or falling asleep (1).

#### Stimuli and experimental conditions

The cue figures used in Experiment 1 were the same as those used in Shirai and Imura’s^6^ Experiment 1: a full-color still image of a young male actor facing toward one of the lateral sides in either a running or a standing position (see Fig. 2). These two images were inspired by a previous study with adults (Gervais *et al.*)^27^. Before posing for pictures, the model viewed the running and standing figures used by Gervais *et al.*^27^. He was then asked to mimic the actions and postures depicted in those figures as closely as possible. To this end, we also instructed him to perform the running action slowly and in an exaggerated way. Hence, the configuration of the running cue figures might differ slightly from that of natural running action, although the model was actually running at the moment the photo was taken. The running figure was 8.4 deg × 15.0 deg, and the standing one was 3.5 deg × 16.6 deg. Each infant was exposed to two experimental conditions: the running condition, which included a running figure as the cue, and the standing condition, which included a standing figure as the cue. All infants included in the final samples completed both the running-condition session and the standing-condition session (20 trials for each session).

### Results and Discussion

**Figure 3:**
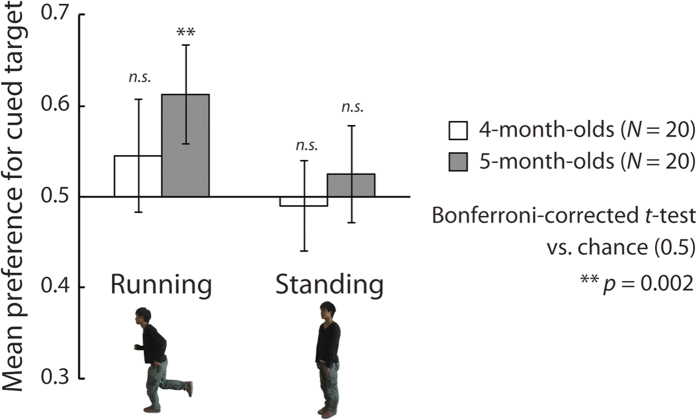
Results of Experiment 1. The vertical axis represents the mean visual preference for the direction cued by the figure. White and dark bars show the mean preferences of the 4- and 5-month-old infants, respectively. Error bars indicate 95% confidence intervals.

Infants’ mean preference scores under each experimental condition are presented in Fig. 3. Bonferroni-corrected two-tailed *t*-tests versus the level of chance (0.5) revealed that the 5-month-old infants showed a significantly greater than chance visual preference for the cued direction under the running condition (*t*(19) = 4.31, *p* = 0.002, *d* = 0.96), whereas they did not show a significant preference under the standing condition (*t*(19) = 0.98, *p* = 0.239, *d* = 0.22). The 4-month-olds showed no significant preference under either the running or the standing condition (*t*(19) = 1.52, *p* > 0.250, *d* = 0.34; *t*(19) = −0.42, *p* > 0.250, *d* = −0.09). Moreover, we performed Bonferroni-corrected binomial tests on the ratio between the proportion of infants with preference scores greater than chance (0.5) and the proportion of those with preference scores less than chance (≤0.5) in each experimental condition. The binomial tests revealed that, under the running condition, the proportion of 5-month-old infants with preference scores greater than 0.5 was significantly (*p* < 0.05) higher than with preference scores less than 0.5 (Table 1). Interestingly, the 4-month-old infants showed no preference in the running condition, but showed a significant ‘negative’ preference in the standing condition (*p* < 0.01), that is, they tended to prefer the direction opposite to that of the standing figure. The negative preference suggests that young infants tend to respond to local visual features rather than configural information in a visual stimulus. The effect of local visual features on the looking behavior of young infants is discussed in the Supplementary Analysis and General Discussion sections. We also conducted a mixed-design two-way analysis of variance (*ANOVA*; infant age [4/5] vs. cue type [run/stand]). The main effects of age (*F*(1,38) = 5.66, *p* = 0.022, *η*_*p*_^2^ = 0.13) and cue type (*F*(1,38) = 5.50, *p* = 0.024, *η*_*p*_^2^ = 0.13) were significant. In contrast, the interaction between the two factors was not significant (*F*(1,38) = 0.29, *p* = 0.596, *η*_*p*_^2^ = 0.01).

The results replicated those reported by Shirai and Imura^6^ in that infants aged at least 5 months of age showed a significant preference for the direction cued by a running but not by a standing figure. Additionally, no significant positive preference for cued direction was observed in the 4-month-old infants under either the running or the standing condition in Experiment 1. This suggests that younger infants may not be able to perceive implied motion from static images. In contrast, the significant main effects of age and cue type revealed by the ANOVA indicated that the preference for the cued direction develops between 4 and 5 months of age, whereas both age groups showed a more pronounced preference for the direction cued by the running than the standing figure. Moreover, the lack of a significant interaction between the two factors implies that, although the younger infants have a less pronounced visual preference for the cued direction than the older infants, the two groups tended to respond similarly to the conditions. Thus, the results of the ANOVA did not provide direct support for the view that younger infants are less able to perceive implied motion than older infants. This seems to be inconsistent with the results of the statistical analyses with regard to differences from chance. This inconsistency suggests that the ability to perceive implied motion develops relatively slowly and continuously and is characterized by substantial individual differences around 4–5 months of age. We will return to this issue in the Supplementary Analysis and General Discussion.

In Experiment 1, we used the standing figure as a control stimulus, as it does not represent any dynamic action on the part of the model. In the next experiment, we used another control stimulus, an inverted running figure, to test infants’ ability to perceive implied motion. In general, perceptions of the structure of the human body^28^ or of human action^29^ can encounter interference when relevant visual stimuli are displayed upside down. The perceived speed of the implied actions associated with static figures is also reduced when such figures are presented upside down^30^. This “inversion effect” has been observed in infants’ implied motion perception, as the preference of 5–8-month-old infants for a direction cued by a running figure was reduced to around the level of chance when an inverted running figure was used as a cue^6^. We tested whether the inversion effect on implied motion perception would be observed in 4- and 5-month-olds in Experiment 2.

## Experiment 2

### Method

#### Participants

The data were obtained from 20 4-month-old (mean age = 123.5 days, SD = ±7.9 days) and 20 5-month-old (mean age = 154.7 days, SD = 8.5 days) infants. All infants were healthy, full-term babies and weighted >2,500 g at birth. An additional five infants also participated in the experiment but were not included in the final sample because they could not complete all experimental trials due to crying (3) or fussing (2).

#### Stimuli and experimental conditions

The experimental method was basically the same as that of Experiment 1 with the exception that the standing figure was replaced with an inverted running figure (see Fig. 2). Thus, there were two experimental conditions in Experiment 2: the running condition and the inverted running condition. All the infants included in the final samples completed both the running-condition session and the inverted running-condition session (20 trials for each session).

### Results and Discussion

**Figure 4:**
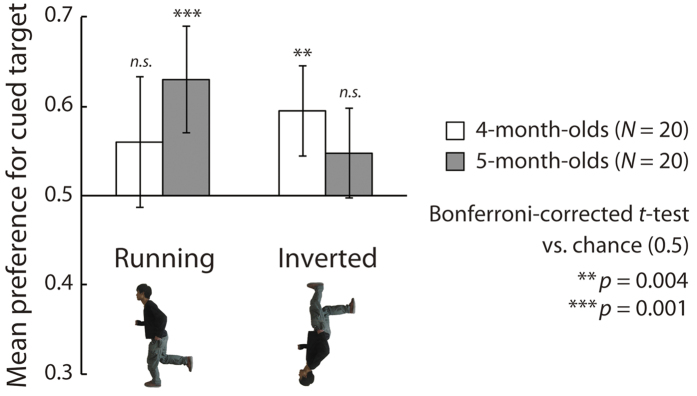
Results of Experiment 2. The vertical axis represents the mean visual preference for the direction cued by the figure. White and dark bars show the mean preferences of the 4- and 5-month-old infants, respectively. Error bars indicate 95% confidence intervals.

Bonferroni-corrected two-tailed *t*-tests versus chance (0.5) indicated that 5-month-old infants showed a significant visual preference for the cued direction under the running condition (*t*(19) = 4.54, *p* = 0.001, *d* = 1.01), whereas they did not show a significant preference under the inverted running condition (*t*(15) = 1.99, *p* = 0.233, *d* = 0.44). In contrast, the 4-month-olds showed a significant preference for the cued direction under the inverted running condition (*t*(19) = 3.91, *p* = 0.004, *d* = 0.87) but not under the running condition (*t*(19) = 1.72, *p* > 0.250, *d* = 0.38) (Fig. 4). Bonferroni-corrected binomial tests of the ratio between the proportion of infants with preference scores greater than chance (0.5) to those of infants with preference scores less than chance (≤0.5) in each experimental condition revealed the same trend as the *t*-tests: the 5-month-old infants showed a significant preference for the standing condition, whereas the 4-month-old infants showed a significant preference for the inverted running condition (*ps* < 0.001; Table 1). A mixed-design ANOVA (infant age [4/5] vs. cue type [running/inverted running]) revealed that the interaction between the two factors was significant (*F*(1, 38) = 4.92, *p* = 0.037, *η*_*p*_^2^ =0.11). The main effects of age (*F*(1, 38) = 0.14, *p* > 0.250, *η*_*p*_^2^ = 0.00) and cue type (*F*(1, 38) = 0.80, *p* > 0.250, *η*_*p*_^2^ = 0.02) were not significant. A simple main effect of cue type was significant among the older infants (*F*(1, 38) = 4.85, *p* = 0.034, *η*_*p*_^2^ = 0.11).

The 5-month-olds showed a significant preference under only the running condition, and the difference in the preference scores under the running condition, which might represent implied action, and the inverted running condition, which might not represent implied action, was significant. These results seem to be straightforward: the 5-month-olds shifted their visual attention toward the direction of the running figure, which might represent implied action, but not toward that of the inverted running figure, which might not represent implied action. In contrast, the 4-month-olds showed a significant preference under only the inverted running condition. Moreover, the ANOVA revealed no significant difference between preferences under the running and the inverted running conditions among the younger infants. These results are somewhat complex. Although the 4-month-olds’ preference was significant under only the inverted running condition, their visual preferences under the running and inverted running conditions were, in some respects, comparable.

One possible explanation for the results for the 4-month-olds is that their visual attention was attracted by local visual information, such as the salient body parts of the model, rather than by the direction of the implied action represented by the configural information about posture. If the younger infants’ visual attention were affected by a particular, salient body part, their visual preference for the cued direction would be relatively irrelevant to the upright/inverted orientation of a cue figure.

Moreover, the 4-month-olds’ significant visual preference under the inverted running condition would be explained by a tendency to be attracted by something in the model’s upper body. It has been reported that the lower visual field is advantageous for detecting objects insofar as it can be compared with the upper visual field^31^. If the younger infants were attracted to the head, nose, eyes, thrusting hand, or some other parts in the upper body of the model, they may have paid more attention to such parts when they appeared in an inverted rather than in an upright running figure. To examine this possibility, we conducted Experiment 3a.

## Experiment 3a

Two new cue figures, i.e., upper-original and lower-original figures (Fig. 2), were reproduced from the original running figure to test the potential effect of the local visual properties of the running figure on infants’ visual preference. We cut and divided the original running figure into two parts, the upper and lower halves, and combined each part with a symmetric replacement of the missing half. If young infants tend to be attracted by salient body parts in the upper body area that are presented in their lower visual field, as we hypothesized, their visual preference would be biased toward the direction cued by the lower part (i.e., the inverted upper body parts) of the upper-original figure. On the other hand, the lower-original figure would not elicit any particular visual preference, because the figure did not contain any components of the upper body in the upper or lower visual field.

### Methods

#### Participants

The data were obtained from 20 4-month-old (mean age = 126.0 days, SD = ± 8.8 days) and 20 5-month-old (mean age = 153.5 days, SD = ± 8.3 days) infants. All infants were healthy, full-term babies and weighed >2,500 g at birth. An additional six infants also participated in the experiment but were not included in the final sample because they could not complete all experimental trials due to crying (5) or inattention to visual stimuli (1).

#### Stimuli and experimental conditions

Two experimental conditions with two new cue figures (upper-original [8.2 deg × 15.0 deg] and lower-original [8.6 deg × 15.0 deg] figures) were included in this experiment. All the infants included in the final samples completed both the upper-original-condition session and the lower-original-condition session (20 trials for each session). Other experimental methods were the same as those in Experiments 1 and 2.

### Results and Discussion

**Figure 5:**
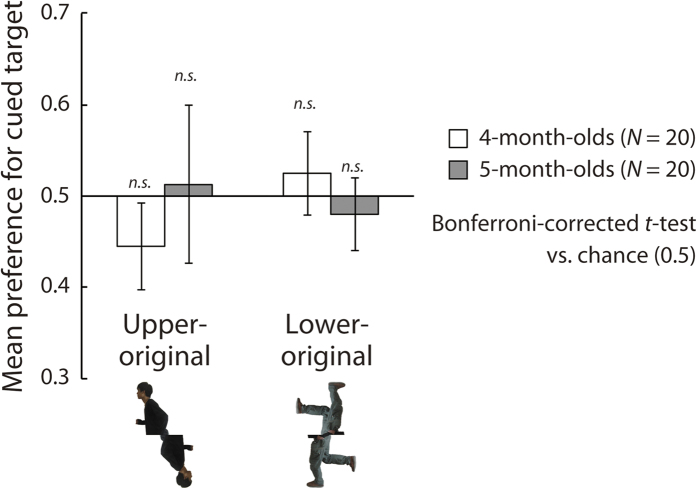
Results of Experiment 3a. The vertical axis represents the mean visual preference for the direction cued by the figure. White and dark bars show the mean preferences of the 4- and 5-month-old infants, respectively. Error bars indicate 95% confidence intervals.

The mean preference score for the direction cued by the original body part in each figure is shown in Fig. 5. Under the upper-original condition, the mean preference score was greater (or less than) than chance when the infants shifted their gaze toward the direction cued by the upper (or lower) part of the cue figure. Thus, if the younger infants were most sensitive to salient visual features of the upper body presented in the lower visual field as hypothesized, they would show a negative preference in the upper-original condition. Under the lower-original condition, the preference score was greater (or less than) than chance when the infants shifted their gaze toward the direction cued by the lower (or higher) part of the cue figure. Thus, if the younger infants participated in Experiment 2 were most sensitive to salient visual features of the upper body presented in the lower visual field, no significant preference would be observed under the lower-original condition in this experiment.

Although 4-month-old infants showed a negative preference for the upper-original condition as expected, the Bonferroni-corrected two-tailed *t*-tests versus chance (0.5) revealed that both age groups showed no significant visual preference under either experimental condition (4 months/upper-original: *t*(19) = −2.40, *p* = 0.111, *d* = −0.54; 4 months/lower-original: *t*(19) = 1.14, *p* > 0.250, *d* = 0.26; 5 months/upper-original: *t*(19) = 0.30, *p* > 0.250, *d* = 0.11; 5 months/lower-original: *t*(19) = −1.05, *p* > 0.250, *d* = −0.23). However, Bonferroni-corrected binomial tests on the ratio between the proportion of infants with preference scores greater than chance (0.5) to those with preference scores less than chance (≤0.5) in each experimental condition revealed that 4-month-olds showed a significant negative preference under the upper-original condition (*p* < 0.05; Table 1), such that they preferred the direction of upper body parts presented in the lower visual field faced. We also conducted a mixed-design two-way ANOVA (infant age [4/5] vs. cue type [upper original/lower original]). The interaction between the two factors was significant (*F*(1, 38) = 4.15, *p* = 0.049, *η*_*p*_^2^ = 0.10), whereas the main effects of age (*F*(1, 38) = 0.16, *p* > 0.250, *η*_*p*_^2^ = 0.00) and cue type (*F*(1, 38) = 0.74, *p* > 0.250, *η*_*p*_^2^ = 0.02) were not significant. Additional analyses indicated that the simple main effects of cue type in the 4-month-olds (*F*(1, 38) = 4.20, *p* = 0.047, *η*_*p*_^2^ = 0.10) was significant and that of infant age under the upper-original condition (*F*(1, 76) = 2.964, *p* = 0.089, *η*_*p*_^2^ = 0.04) was marginally significant.

The fact that the 4-month-olds showed a negative visual preference under the upper-original condition suggests that the younger infants were sensitive to salient body features (e.g., pointing hand or head) in the sensitive visual field (lower visual field). These findings are consistent with those of the inverted-running condition in Experiment 2 in which the younger infants showed a preference for the direction the upper body presented in the lower visual field faced. Additionally, the younger infants demonstrated significantly different visual preferences for the upper-original and lower-original conditions, whereas the older infants did not show such a difference. This implied that the visual preferences of the younger and older infants differed. It is possible that the visual attention of the younger infants was affected by local cues in the cue figures, whereas that of the older infants was not. Thus, the 4-month-olds’ visual preference for the cued direction depicted by the running figure found in Experiments 1 and 2 might have been due to the local visual properties of the figures rather than to configural postural information. It may be that by the age of 5 months, human infants are more sensitive to implied dynamic actions extracted from configural information than salient local visual information, such that the representation of the implied motion supersedes attention to salient local visual information.

## Experiment 3b

We conducted a follow-up experiment to clarify the results of Experiment 3a, to test whether the infants who participated in that experiment showed a significant preference for the original running figure. The aim of this experiment was to explore the effect of the configural postural information provided by the figure in the same infants who participated in Experiment 3a.

### Methods

#### Participants

The infants who participated in all experimental trials in Experiment 3a took part in this experiment after completing Experiment 3a. Because six younger and two older infants could not complete all trials of this experiment due to crying (6) and inattention to visual stimuli (2), the final sample was composed of 14 4-month-old (mean age = 126.5 days, SD = ±8.6 days) and 18 5-month-old (mean age = 154.2 days, SD = ±8.4 days) infants.

#### Stimuli and experimental conditions

The experimental method and procedure was the same as those under the running condition in Experiment 1. All the infants included in the final samples completed the running-condition session (20 trials).

### Results and Discussion

**Figure 6:**
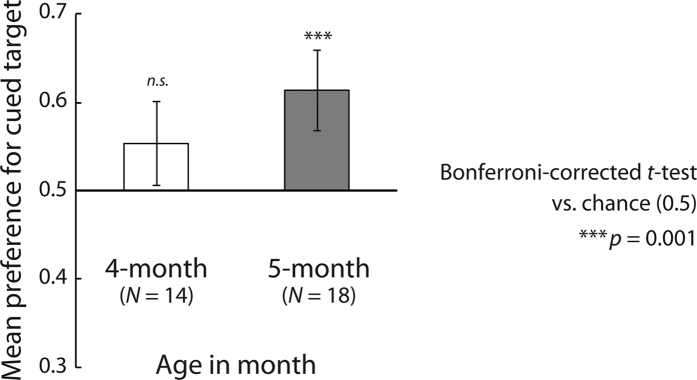
Results of Experiment 3b. The vertical axis represents the mean visual preference for the direction cued by the cue figure. Error bars indicate 95% confidence intervals.

Only the older group showed a significant preference for the cued direction (Bonferroni-corrected two-tailed *t*-tests versus chance (0.5); 4 months: *t*(13) = 1.98, *p* = 0.122, *d* = 0.53; 5 months: *t*(17) = 4.26, *p* = 0.001, *d* = 1.00) (Fig. 6). Bonferroni-corrected binomial tests on the ratio between the proportion of infants with preference scores greater than chance (0.5) to those with preference scores less than chance (≤0.5) revealed similar trends to the *t*-tests in that the 5-month-old (*p* < 0.01) but not 4-month-old infants showed a significant preference under the running condition (Table 1). On the other hand, a two-tailed *t*-test revealed no significant difference in the visual preference of the younger and older infants (*t*(30) = 1.56, *n.s.*, *d* = 0.44). These results perfectly replicated those obtained under the running condition in Experiment 1.

## Supplementary Analysis

A possible limitation of our study is that the failure to find a significant age-related difference in the preferences for running direction was due to our relatively small sample size. Therefore, we created a larger sample by collapsing the data obtained under the running conditions in Experiments 1, 2, and 3b and re-analyzed the preferences of the two groups. The supplementary analysis included the data of 54 4-month-old infants (mean age = 124.5 days, SD = 8.6) and those of 58 5-month-old infants (mean age = 154.6 days, SD = 8.5).

### Results and Discussion

Bonferroni-corrected two-tailed *t*-tests versus chance (0.5) revealed a significant visual preference under the running condition in both age groups (4-month-olds: *t*(53) = 2.92, *p* = 0.014, *d* = 0.40; 5-month-olds: *t*(57) = 7.68, *p* < 0.001, *d* = 1.01). Conversely, Bonferroni-corrected binomial tests on the ratio between the proportion of infants with preference scores greater than chance (0.5) to those with preference scores less than chance (≤0.5) revealed a significant preference only in the 5-month-old infants (*p* < 0.001; Table 1). This contradictory finding suggest that the preference for direction of an implied running action was less stable or robust in the younger than in the older infants. Moreover, although the supplementary analysis revealed a partially significant preference for implied direction in the younger infants, the analyses using the smaller sample (Experiment 1, 2 and 3a) revealed no significant preference in this group. We postulate two possible explanations for this disparity. First, the sensitivity to implied motion may have been weak or highly variable in the young infants, and thus, the small sample size may not have had sufficient power to detect significant differences. Alternatively, the significant effect revealed that using a larger sample may reflect a preference for local visual properties of the figure rather than an implied action perceived from the configural shape of the running figure. Inverted figures tend to weaken the perception of dynamic action^30^; therefore, if the younger infants were able to perceive implied motion from the running figure, their visual preference for the direction of the running figure should have been the lowest under the inverted condition. On the contrary, the young infants showed a significant preference under the inverted condition (Experiment 2) and a positive, but non-significant preference under the upright conditions (Experiments 1 and 2). It may be that the young infants were attracted to salient features of the upper body of the running figure, and that preference may have been bolstered by presentation of those features in the lower visual field (i.e., the inverted running condition in Experiment 2), the more sensitive part of the visual field. In contrast, the preference for the upright running figure, in which the local salient features were presented in the upper visual field, may have been too weak to reach significance in the analysis using the smaller sample size, but strong enough to be significant in the collapsed data analysis. Our results do not allow us to determine which explanation is more likely. However, the latter explanation (which involves basic visual functions such as detection of local visual saliency) is based on a less complex hypothetical mechanism than the former (which involves higher visual functions such as extraction of dynamic representation from global form information). We conclude that the significant preference revealed by the supplementary analysis stemmed from the young infants’ sensitivity to salient local visual cues in the running figure. Thus, our findings suggest that the qualitative aspect of the preference for the implied running action differed between the younger and the older infants.

Furthermore, we found a quantitative difference in the preferences between the younger and the older infants. A two-tailed *t*-test revealed a significant difference in the preference for the running direction between the age groups (*t*(110) = 2.79, *p* = 0.006, *d* = 0.47). The fact that this difference was significant only in the analysis using the relatively large sample suggests that the effect was modest. However, a direct comparison of the younger and the older age groups revealed a significant developmental difference in the preference for the figure, which implies dynamic running action. We speculate that the presence of two confounding factors (local visual saliency vs. configural visual information representing a dynamic action) may have driven the preference in the direction of the running figure in both groups; thus the analysis with the smaller sample size did not yield a significance difference between groups. It may be that the younger infants were more sensitive to salient local visual cues, whereas the older infants were more sensitive to the configural visual information. Because local and configural visual information can drive the infants’ preference in a direction consistent with the running figure, it is likely that a difference in the magnitude of the apparent preference may be smaller and non-significant in a relatively small sample.

## General Discussion

In summary, the 5-month-old infants showed a significant visual preference for the cued direction only when they were presented with the running figure, which might represent the dynamic running action of a model (Experiments 1, 2, and 3b). This means that the older infants’ visual preference was based on the configural postural information contained in the running figure. In contrast, the 4-month-olds showed no significant visual preference for the direction cued by the running figure (Experiments 1, 2, and 3b). Instead, they showed a significant preference only under the inverted running condition, and the size of the preference was not significantly different from that elicited under the upright running condition (Experiment 2). Moreover, the younger infants’ visual preference seems to have been modulated by local visual information rather than the configural postural information in the cue figures (Experiment 3a). These results suggest that the observed preference for the direction cued by the running figure qualitatively differed between 4- and 5-month-olds; the older infants responded to the configural postural information of the figure, which may have represented the implied action of the model, whereas the younger infants responded to local visual properties, which may not have represented the implied action of the model in the figure (see the Results and Discussion of the Supplementary Analysis). Hence, we concluded that the older, but not the younger, infants perceived implied motion from the running figure and that implied motion perception emerges between 4 and 5 months of age.

As mentioned in the Introduction, implied motion perception is thought to be related to the functions of both the ventral and dorsal pathways, which are related to the processing of visual form and motion, respectively. The development of implied motion perception observed in the present study might reflect the maturation of interactions between these cortical pathways. Significant sensitivity to global form information emerges around 5 months of age, whereas sensitivity to global motion information emerges around 3 months of age^15^. It is plausible that functional interactions between the two visual pathways appear after the independent maturation of each of these pathways. The ages of the infants who participated in the present study (i.e., 4 and 5 months) might be consistent with the developmental period in which the functional interactions between the ventral and dorsal pathways emerge. It should be noted that another type of form–motion interaction, the shape perception of an object moving behind a slit (slit-viewing^32^), also develops between 3–4 and 5–6 months of age (Imura and Shirai, 2014)^32^. The results reported by Imura and Shirai^33^ and those of the present study imply that the fundamental interactive functions between the ventral and dorsal pathways emerge around 4–5 months of age in human infants.

In general, we conclude that implied motion perception, the ability to perceive dynamic directional events from still images, develops between 4 and 5 months of age that the neural bases for the development of implied motion may be the interactive structures that connect the ventral and dorsal cortical pathways, which may mature by 5 months of age.

## Additional Information

**How to cite this article**: Shirai, N. and Imura, T. Emergence of the ability to perceive dynamic events from still pictures in human infants. *Sci. Rep.* **6**, 37206; doi: 10.1038/srep37206 (2016).

**Publisher’s note**: Springer Nature remains neutral with regard to jurisdictional claims in published maps and institutional affiliations.

## Figures and Tables

**Table 1:** Ratio of the proportion of infants with a greater than chance (0.5) preference for the cued direction to the total number of participants in Experiments 1, 2, 3a, 3b (Supplementary Analysis).

	Age	Figure conditions				
		Running	Standing	Inverted running	Upper original	Lower original
Experiment 1	4 months	8/20	4/20	n/a	n/a	n/a
	5 months	15/20	10/20	n/a	n/a	n/a
Experiment 2	4 months	11/20	n/a	17/20	n/a	n/a
	5 months	17/20	n/a	10/20	n/a	n/a
Experiment 3a	4 months	n/a	n/a	n/a	5/20	12/20
	5 months	n/a	n/a	n/a	11/20	6/20
Experiment 3b	4 months	9/14	n/a	n/a	n/a	n/a
	5 months	14/18	n/a	n/a	n/a	n/a
Extra analysis	4 months	27/54	n/a	n/a	n/a	n/a
	5 months	46/58	n/a	n/a	n/a	n/a
